# Proteomic Investigations of Proteases Involved in Cotyledon Senescence: A Model to Explore the Genotypic Variability of Proteolysis Machinery Associated with Nitrogen Remobilization Efficiency during the Leaf Senescence of Oilseed Rape

**DOI:** 10.3390/proteomes5040029

**Published:** 2017-11-02

**Authors:** Marine Poret, Balakumaran Chandrasekar, Renier A. L. van der Hoorn, Laurent Coquet, Thierry Jouenne, Jean-Christophe Avice

**Affiliations:** 1 Université de Caen Normandie, Normandie Université, F-14032 Caen, France; marine.poret@unicaen.fr; 2 UCN, UMR INRA–UCBN 950 Ecophysiologie Végétale, Agronomie & Nutritions N.C.S., FED 4277 Normandie Végétal, F-14032 Caen, France; 3 INRA, UMR INRA–UCBN 950 Ecophysiologie Végétale, Agronomie & Nutritions N.C.S., FED 4277 Normandie Végétal, F-14032 Caen, France; 4 The Plant Chemetics Laboratory, Department of Plant Sciences, University of Oxford, South Parks Road, Oxford OX1 3RB, UK; balakumaran.chandrasekar@plants.ox.ac.uk (B.C.); renier.vanderhoorn@plants.ox.ac.uk (R.A.L.v.d.H.); 5 The Plant Chemetics Laboratory, Max Planck Institute for Plant Breeding Research, Carl-von-Linne Weg 10, 50829 Cologne, Germany; 6 CNRS UMR6270, “PISSARO” Facilities of IRIB-HN, Faculté des Sciences de Rouen, F-76821 Mont-Saint-Aignan, France; laurent.coquet@univ-rouen.fr (L.C.); Thierry.jouenne@univ-rouen.fr (T.J.)

**Keywords:** *Brassica napus* L., protease activity, senescence, cotyledon, nitrogen remobilization efficiency, phytohormones, genotypic variability

## Abstract

Oilseed rape is characterized by a low nitrogen remobilization efficiency during leaf senescence, mainly due to a lack of proteolysis. Because cotyledons are subjected to senescence, it was hypothesized that contrasting protease activities between genotypes may be distinguishable early in the senescence of cotyledons. To verify this assumption, our goals were to (i) characterize protease activities in cotyledons between two genotypes with contrasting nitrogen remobilization efficiency (Ténor and Samouraï) under limiting or ample nitrate supply; and (ii) test the role of salicylic acid (SA) and abscisic acid (ABA) in proteolysis regulation. Protease activities were measured and identified by a proteomics approach combining activity-based protein profiling with LC-MS/MS. As in senescing leaves, chlorophyll and protein contents decrease in senescing cotyledons and are correlated with an increase in serine and cysteine protease activities. Two RD21-like and SAG-12 proteases previously associated with an efficient proteolysis in senescing leaves of Ténor are also detected in senescing cotyledons. The infiltration of ABA and SA provokes the induction of senescence and several cysteine and serine protease activities. The study of protease activities during the senescence of cotyledons seems to be a promising experimental model to investigate the regulation and genotypic variability of proteolysis associated with efficient N remobilization.

## 1. Introduction

Oilseed rape (*Brassica napus* L.) is the second largest oleaginous crop worldwide behind soybean with a world production attaining about 64 million tonnes of grain [1] and the production of oilseed rape is experiencing a resurgence of interest thanks to the development of many food processing, green chemistry, and industrial markets based on biosourced products. However, despite a strong need for N fertilizers [2], oilseed rape is characterized by a weak N use efficiency with only 50% of N from fertilizers being recovered in seeds [3]. This low N use efficiency is mainly related to a low N remobilization efficiency during the ‘sequential’ leaf senescence that occurs during the vegetative stages and the transition between vegetative and reproductive phases of development [4,5,6,7,8,9]. Considering that the improvement of N remobilization efficiency is a major lever to optimize the agri-environmental performance of oilseed rape, it is necessary to understand the N remobilization associated with senescence [7,8,9,10].

Senescence is a genetically controlled process involving many physiological, biochemical, and molecular events, resulting in a strong degradation of macromolecules as nucleic acids, lipids, and proteins, and leading to the death of all or part of the plant [7,11,12]. ‘Sequential’ senescence, affecting older leaves along the axis of the plant, leads to nutrient remobilization from source leaves to the sink organs such as young leaves [7]. This process is not only a degenerative process, but is an essential process for the survival of the rest of the plant and the survival of the species *via* seed production [13]. Moreover, senescence is a process specifically linked to seed yield and plant productivity [14,15,16].

The senescence process is tightly controlled by environmental and endogenous factors [17,18]. Among the endogenous factors, many hormonal pathways are involved in the negative or positive regulation of leaf senescence. These pathways play a role at all stages of senescence, whether they be the initiation, progression, or final stage of senescence [18,19,20,21,22,23]. Cytokinins (CKs), gibberellins (GAs), and auxin (IAA) are hormones involved in the negative regulation of senescence [20,24,25,26,27]. In contrast, abscisic acid (ABA), salicylic acid (SA), ethylene (ET), methyl jasmonate (MeJA), brassinosteroids (BR), and strigolactones (SL) have the capacity to positively regulate senescence [18,19,20,21,23].

During senescence, protein degradation represents the most important degradation process and N remobilization efficiency improvement in oilseed rape is tightly associated with the optimization of proteolysis during leaf senescence [8,9,28,29]. Recent work has focused on the understanding of proteolysis processes during the senescence of oilseed rape to improve the N remobilization efficiency [7,9,29]. During leaf senescence, protein breakdown is related to many protease classes: aspartic proteases, metalloproteases, serine proteases (SPs), cysteine proteases (CPs), and the proteasome [30]. Among these classes, CPs and SPs are the most strongly associated with leaf senescence in various species [30,31,32,33,34]. Furthermore, in oilseed rape, recent studies have shown that the genotypic variability of leaf N remobilization efficiency under N limitation is related to the proteolysis efficiency and specific protease activities [8,9]. For instance, under low N conditions, the genotype Ténor characterized by a high N remobilization efficiency was able to maintain its leaf biomass production thanks to higher soluble protein degradation and N remobilization, contrary to the genotype Samouraï, which is characterized by a low N remobilization efficiency [8]. This higher leaf proteolysis of Ténor compared with Samouraï was correlated with an increase in SP and CP activities and with the appearance of new CP activities (RD21-like, RD19-like, SAG12-like, cathepsin-B, XBCP3-like, and Aleurain-like proteases) [35]. Additionally, compared with Samouraï, the genotype Ténor was characterized by a higher hormonal ratio ([SA] + [ABA])/([CK]) during leaf senescence that was induced by nitrate limitation. These changes in the ([SA] + [ABA])/([CK]) ratios are also associated with a higher proteolysis and the increase or the induction of protease activities [35]. Based on these previous results and on the fact that cotyledons are also subjected to a senescence process, it could be assumed that the contrasting protease activities observed during leaf senescence at the vegetative stages between genotypes may also be distinguishable early in the senescence of cotyledons. To verify this hypothesis, our aims were (i) to characterize senescence-associated protease activities of Ténor and Samouraï during the senescence of cotyledons; and (ii) to validate the putative role of SA and ABA in the regulation of protease activity during the senescence of cotyledons in oilseed rape. In order to reach these goals, CP and SP protease activities were measured and identified by activity-based protein profiling (ABPP) and LC-MS/MS analysis during the senescence of cotyledons under high or low N conditions or after SA and ABA infiltrations.

## 2. Materials and Methods

### 2.1. Chemicals

Diisopropylfluorophosphate (DFP) and E-64 were provided by SIGMA-ALDRICH^®^. The probes MV201, DCG04, FP-Rh, and FP-biotin [36,37] were kindly given by Professor Renier van der Hoorn (The Plant Chemetics Laboratory, University of Oxford, Oxford, UK).

### 2.2. Experimental Design

#### 2.2.1. Study of Cotyledon Senescence under High N or Low N Conditions

Seeds of two genotypes of *Brassica napus* L. (cv. Ténor and Samurai) were provided by the biological resource center Bracysol (Le Rheu, France). Seeds were sown and supplied with 25% Hoagland nutrient solution ((1.25 mM Ca(NO_3_)_2_·4H_2_O, 1.25 mM KNO_3_, 0.5 mM MgSO_4_, 0.25 mM KH_2_PO_4_, 0.2 mM EDTA·2NaFe·3H_2_O, 14 μM H_3_BO_3_, 5 μM MnSO_4_, 3 μM ZnSO_4_, 0.7 μM (NH_4_)_6_Mo_7_O_24_, 0.7 μM CuSO_4_, 0.1 μM CoCl_2_) to obtain seedlings with mature cotyledons (15 days). Seedlings were subjected to a photoperiod of 16 h (20 °C (day)/15 °C (night)) and received 400 μmoles photon. s^−1^·m^−2^ of photosynthetically active radiation. After 15 days, corresponding to the beginning of treatments (Day 0 (D0)), cotyledons were separated into two sets and were supplied with 25% Hoagland solution containing two different concentrations of nitrate: high (HN: 3.75 mM of Ca(NO_3_)_2_·4H_2_O) or low nitrate (LN: 0.375 mM Ca(NO_3_)_2_·4H_2_O with Ca and K compensated with addition of 1.25 mM CaCl_2_·2H_2_O and 0.875 mM KCl) for five days. Chlorophyll content was measured at Day 0 (D0), 2 (D2), and 5 (D5) with a SPAD meter (Soil Plant Analysis Development; Minolta, SPAD-502 model). As previously described in several plants and in oilseed rape [6,8,9], the measurement of leaf transmittance performed by the SPAD meter is directly related to the content of chlorophylls.

The cotyledons were harvested at Day 0 and after five days of treatment (D5). For each date and each treatment, three biological replicates (one replicate comprising six cotyledons) were harvested and the samples were immediately frozen in liquid nitrogen before storage at −80 °C before further analyses.

#### 2.2.2. Effect of the Phytohormone Infiltrations on the Senescence of Cotyledons

Seeds of *Brassica napus* L. (cv. Ténor) were sown and cultivated for 15 days under the same conditions described above (Section 2.2.1). After 15 days, corresponding to the beginning of the experiment (Day 0 (D0)), three treatments were applied. Cotyledons were infiltrated with either 1 mL of water, salicylic acid (SA; 500 μM), or abscisic acid (ABA; 50 μM). The infiltration was performed on the abaxial surface of the cotyledon with a needleless syringe by simple pressure. The infiltration was repeated on the same cotyledons, 6 and 12 h after the first injection.

The cotyledons were harvested at D0 and after three days of treatment (D3). Before harvest, the chlorophyll content was measured at D0 and D3 with a chlorophyll meter (SPAD-502). The number of replicates and the conditions of harvest and sample storage were similar to those described above (Section 2.2.1).

### 2.3. Detection and Identification of Active Proteases

Soluble proteins were extracted from 200 mg of frozen cotyledons with 1 mL of water. After centrifugation (10 min, 13,000 *g*, 4 °C), proteins were quantified in equivalent bovine serum albumin (BSA) by protein-dye staining [38].

The detection of active proteases was performed with protease labeling according to Poret et al. (2016) [29]. Protein extracts (20 μg) were incubated in a mix final volume of 200 μL containing 0.5 μM of MV201, 50 mM of sodium acetate buffer (pH 5.5), and 2 mM dithiothreitol. In parallel, 20 μg of protein extract was incubated in a mixed final volume of 200 μL containing 0.5 μM of FP-Rh and 150 mM Tris-base buffer (pH 7.5). The two different mixtures were incubated for 4 h (MV201) or 1 h (FP-Rh) in the dark under gentle agitation.

As a control, a mixture of equal volumes of soluble protein extracts from cotyledons at D0 and D5 treated under HN or LN conditions or D0 and D3 treated with phytohormones were prepared and 20 μg of each mixture was treated as described above. In the no-probe-control, an equal volume of DMSO was added. Competition experiments were also carried out by performing a pre-treatment for 30 min with 50 μM of E-64 (competition with MV201) or DFP (competition with FP-Rh) before adding probes. The addition of 1 mL of ice-cold acetone stopped the reaction by the precipitation of proteins. After centrifugation (15 min, 16,000 *g*, 4 °C), the pellet was dissolved in 2× SDS-PAGE gel-loading buffer (140 mM sodium dodecyl sulfate, 200 mM Tris, 20% glycerol, 5% β-mercaptoethanol, 0.3 mM Bromophenol Blue), and heated at 90 °C for 10 min. The protein samples were loaded (20 μg per lane) and separated on 12% SDS-PAGE gels. The fluorescently labeled proteins were detected by a ProXPRESS 2D proteomic Imaging System (PerkinElmer, Villebon sur Yvette, France) with excitation wavelength at 532 nm and emission wavelength at 580 nm. Signals were quantified with ImageJ software. Gels were stained with Coomassie Brilliant Blue stain (0.5 g CBB G250, 10% acetic acid, 45% methanol in ultra-pure water (*v*/*v*)), destained (10% acetic acid, 40% methanol in ultra-pure water (*v*/*v*)), and scanned to control the protein quantity after electrophoresis.

To identify previously detected active proteases, protein extracts were labeled with the biotinylated-tagged probes, DCG04 (PLCP labeling) or FP-biotin (SH labeling), and pull-downs of biotinylated proteins were performed with a modified protocol from Poret et al. (2016) [29]. In summary, 900 μg of protein was labeled with 10 μM of DCG04 or FP-biotin in labeling buffer (50 mM sodium acetate buffer, pH 5.5, 2 mM DTT) for DCG04 or 50 mM Tris-buffer (pH 7.5) for FP-biotin. Samples were incubated at room temperature under agitation (4 h or 1 h for DCG04 or FP-biotin respectively). Another aliquot was treated as described above but without probes as the control (no probe control). Reactions were stopped and the biotin-proteins were purified using streptavidin beads as described by Chandrasekar et al. (2014) [39]. After separation on 12% SDS-PAGE gels, eluted proteins were detected by silver nitrate staining [40]. Bands of interest were excised manually and the trypsin digestion was performed as described by Girondé et al. (2016) [9].

The protease identification was carried out by the proteomic platform IFR MP 23 at the University of Rouen Normandy (Normandy, France). The protein bands of interest were excised from SDS-PAGE gels and reduced at 50 °C for 1 h with 10 mM dithiothreitol and alkylated for 1 h in the dark with 55 mM iodoacetamide. The gel fragments were washed several times with water and ammonium carbonate, dehydrated with acetonitrile, and dried. Trypsin digestion of gel fragments was performed overnight with a dedicated automated system (MultiPROBE II, PerkinElmer) and followed by two incubations for 15 min in acetonitrile solution to extract peptides from the gel pieces. Peptide extracts were then dried and dissolved in a buffer containing 3% acetonitrile and 0.1% formic acid for chromatographic elution. Peptides were enriched, separated, and analysed using a 6520 Accurate-Mass Q-TOF LC/MS equipped with an HPLC-chip cube interface (Agilent Technologies, Massy, France). The fragmentation data were interpreted using Mass Hunter software (version B.03.01, Agilent Technologies, Les Ulis, France). For protein identification, MS/MS peak lists were extracted, converted into mzdata.xml format files, and compared with the protein database (NCBInr-Viridiplantae) using the MASCOT Daemon search engine (version 2.1.3; Matrix Science, London, UK). The searches were performed with no fixed modification and with variable modifications for the oxidation of methionine, and with a maximum of one missed cleavage site. MS/MS spectra were searched with a mass tolerance of 20 ppm for precursor ions and 0.6 Da for MS/MS fragments. Only peptides matching an individual ion score >41 were considered. Proteins with two or more unique peptides matching the protein sequence were considered as a positive identification. The identified protein with the best match is provided with the UniProt or NCBI/GenBank accession number, while other proteins recognized with the same peptides are also presented. The score, peptide matches, different peptide matches, experimental mass, and theoretical mass are presented. PLCPs and SHs were ranked according to the classification of Richau et al. (2012) [37] and the MEROPS database, respectively.

### 2.4. Statistical Analysis

The Ryan-Joiner test at 95% was used to study the normality of the data. By using Addinsoft^®^ Excel 2010/XLStat^®^ 2016 (Paris, France, the means were compared by analysis of variance (ANOVA) and the Newman-Keuls test. If the normality law of the data was not respected, the non-parametric test of Kruskal-Wallis was used. Statistical significance was postulated at *p* < 0.05. Three biological repetitions were analyzed (*n* = 3, one replicate comprising six cotyledons) for all measurements and all the data are presented as the mean ± standard deviation (SD).

## 3. Results and Discussion

### 3.1. Characterization of Physiological and Proteolysis Events during Cotyledon Senescence in Two Genotypes of Oilseed Rape with Contrasted N Remobilization Efficiency

#### 3.1.1. Physiological Modifications during Cotyledon Senescence

**Figure 1:**
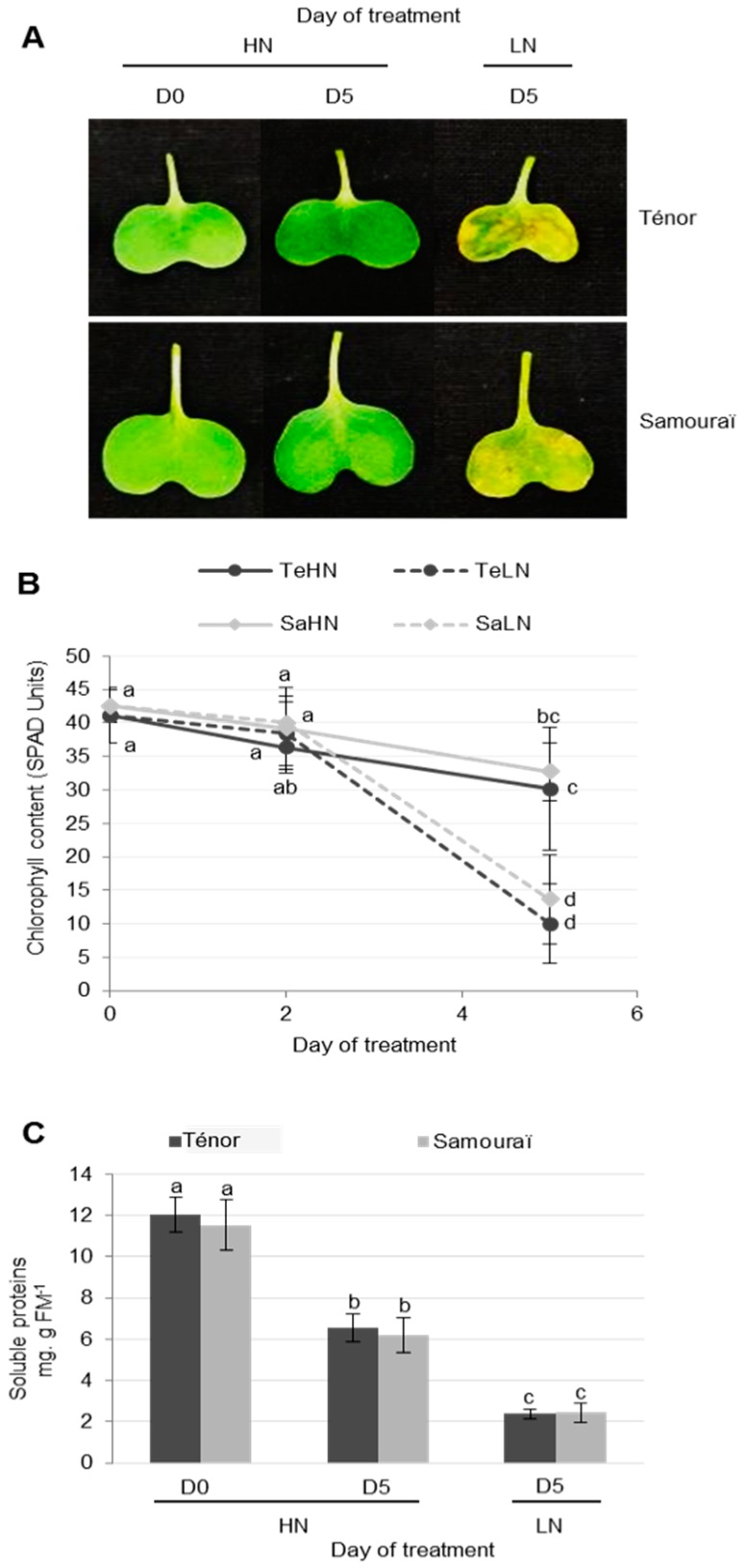
Chlorophyll (**A**,**B**) and soluble protein (**C**) contents during cotyledon senescence in two genotypes of oilseed rape supplied with high (HN) or low (LN) nitrate for five days. Cotyledons (15 days old) of two different genotypes (Ténor and Samouraï) were subjected to ample (HN: 3.75 mM NO_3_^−^) or low nitrogen supply (LN: 0.375 mM NO_3_^−^) for five days. Vertical bars indicate ± SD of the mean (*n* = 3). Different letters indicate statistical differences (*p* < 0.05, ANOVA, Newman-Keuls test).

In order to characterize physiological changes associated with the senescence of cotyledons, 20 day old plants of two genotypes of oilseed rape (cv. Ténor and Samouraï) were subjected to ample (HN: 3.75 mM NO_3_^−^) or low nitrogen supply (LN: 0.375 mM NO_3_^−^) for five days. As expected, the results showed that chlorophyll content decreased during the senescence of cotyledons after five days, regardless of the genotype (Figure 1A,B). In cotyledons subjected to nitrate limitation, the chlorophyll content strongly declined (on average −70%) compared to only −25% in cotyledons supplied with HN solution (Figure 1B). During leaf senescence, it has been demonstrated that the decrease in chlorophyll content is greater in Ténor than in Samouraï [8,9]. In contrast to the leaves, during cotyledon senescence under N limitation, there was no difference in the decline of chlorophyll levels between Ténor and Samouraï (Figure 1B).

The soluble protein contents decreased significantly during cotyledon senescence after five days, particularly under LN treatment (−80%) compared with HN treatment (−46%) (Figure 1C). Nevertheless, this decrease was not significantly different between Ténor and Samouraï. These observations confirm those previously described in cotyledons [41] or leaves [8,9,42] in oilseed rape for total proteins during senescence. However, unlike these observations in cotyledons, it was recently reported that the rate of proteolysis during leaf senescence induced by N limitation was stronger for Ténor than Samouraï [8,9]. Based on these results, it was confirmed that the senescence of cotyledons was accelerated by an N limitation similar to leaf senescence in oilseed rape [41,42]. However, even though Ténor was characterized by a higher N remobilization efficiency than Samouraï during the sequential leaf senescence associated with N limitation [8,9], there was no physiological manifestation of this genotypic difference during cotyledon senescence under our experimental conditions (i.e., after five days of N treatment). This could be due to the fact that the senescence of cotyledons in our experiment was particularly rapid, especially in response to N limitation treatment (Figure 1). This means that it is necessary to have several observations between Day 2 and Day 5 to see if the changes in the chlorophyll content and rates of proteolysis associated with the contrasted N remobilization efficiency between genotypes during leaf senescence were already present in Ténor compared with Samouraï during the senescence of cotyledons.

#### 3.1.2. Modifications of Protease Activities during the Senescence of Cotyledons

Standard protease activity profiling of SHs (using FP-Rh, a specific fluorescent probe of serine hydrolases) and CPs (using MV201, a specific fluorescent probe of papain-like cysteine proteases, PLCPs) was performed on soluble protein extracts from cotyledons after 0 and five days of LN and HN treatments.

**Figure 2:**
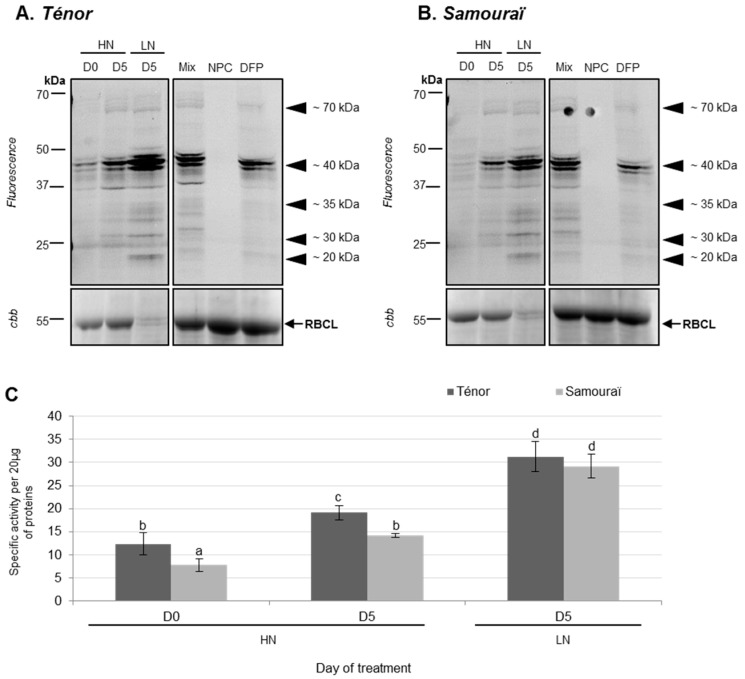
Serine hydrolase (SH) activity at pH 7.5 during cotyledon senescence in two genotypes of oilseed rape supplied with high (HN) or low (LN) nitrate for five days. Soluble proteins of cotyledons were extracted from Ténor or Samouraï plants after 0 and five days of HN (3.75 mM NO_3_^−^) or LN (0.375 mM NO_3_^−^) treatment. Labeling of protease activity with FP-Rh (specific fluorescent probe of SHs) (pH 7.5; 1 h labeling) was performed. After SDS-PAGE, the fluorescence was detected with a scanner (**A**) Ténor; (**B**) Samouraï. As a control, Mix refers to a mix of 0 and five day extracts (HN and LN) in the presence of FP-Rh, NPC (no probe control) refers to the same mix in the absence of FP-Rh, and DFP refers to the same mix in the presence of FP-Rh and DFP (specific inhibitor of serine proteases). Positions of active proteases are represented by black arrowheads. SH global activity characterized by the fluorescence intensity was calculated with the Image J^®^ (**C**) software. The presented gel is representative of three biological replicates. Gel stained with Coomassie brilliant blue (cbb) shows the RuBisCO large sub-unit (RBCL) and the decline of this major protein indicated that proteolysis and N remobilization are induced. Vertical bars indicate ± SD of the mean (*n* = 3). Different letters indicate statistical differences (*p* < 0.05, ANOVA, Newman-Keuls test).

Using the labeling of SHs with FP-Rh, it was observed that the total specific activity of SHs was significantly increased between 0 and five days, particularly under the LN condition for both genotypes (2.5 fold for Ténor and 3.7 fold for Samouraï; Figure 2B). Indeed, activities at ~70, ~40, ~35, ~30, and ~25 kDa increased during senescence between D0 and D5 under LN conditions in Ténor and Samouraï cotyledons (Figure 2A). Activities of SHs were higher in Ténor than Samouraï cotyledons after five days of HN treatment. Finally, the increase in SH activities was significantly correlated with the decrease in soluble proteins shown in Figure 1C (r = −0.899; *p*-value < 0.0001). These results were consistent with previous reports showing that a gene encoding a serine carboxypeptidase was up-regulated gradually from the green to yellow cotyledons of cucumber [43]. In addition, during the leaf senescence of oilseed rape, the global activity of serine proteases is increased, particularly in response to N limitation [9].

**Figure 3:**
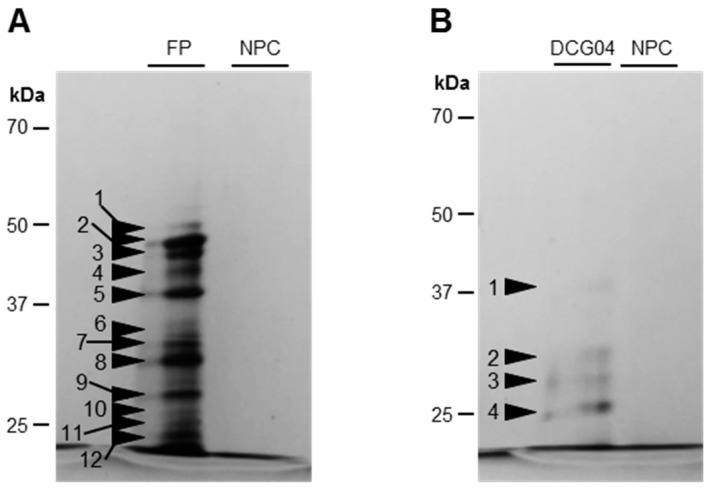
Detection of SHs (**A**) and PLCPs (**B**) labeled with FP-biotin and DCG04 in senescent cotyledons of oilseed rape (cv. Ténor) after five days of LN treatment. To characterize SHs and PLCPs observed in Figure 2 and Figure 4, a pull-down of biotinylated proteins was performed after labeling with the biotin-tagged probes FP-biotin (**A**) and DCG04 (**B**). After SDS-PAGE, proteins were detected by staining with silver nitrate (for details see “Materials and Methods”). NPC refers to the no probe controls. Black arrowheads indicate the excised zones.

To identify labeled SHs (Figure 2), activity-dependent labeling with FP-biotin followed by a pull-down of biotinylated proteins was performed on Ténor cotyledons after five days of LN treatment (Figure 3, Table 1 and Table S1). The labeling allowed the detection of 12 bands of active SHs between 50 and 20 kDa (Figure 3) and the bands were identified by LC-MS/MS (Table 1; Table S1). We identified 14 serine proteases corresponding to 12 carboxypeptidases (S10), one subtilisin-like protease (S8), and one Deg-protease. Furthermore, other SHs were identified, corresponding to four lipases, four carboxyl-esterases, four methyl-esterases, and two thiol-esterases (Table S1). Interestingly, among the 14 identified serine proteases, 10 were also identified as active proteases during the sequential leaf senescence of oilseed rape [29]. As observed for leaf senescence, the activity of SHs was also increased during the senescence of cotyledons associated with LN treatment, regardless of the genotype. Moreover, many activities of serine proteases implicated in the leaf senescence of Ténor were also found during cotyledon senescence. Based on these results, this type of senescence could be a promising model to study proteases associated with leaf senescence. However, the genotypic differences observed between Ténor and Samouraï during leaf senescence were not observed during the senescence of cotyledons. That is why SP activities did not seem to be the best target for distinguishing between genotypes at the cotyledon stage.

**Figure 4:**
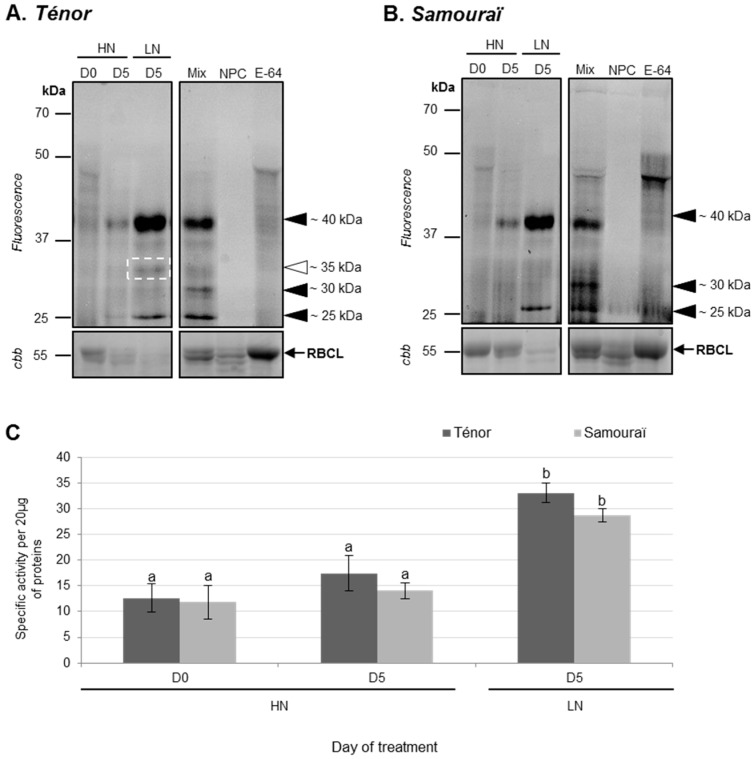
PLCP activities at pH 5.5 during cotyledon senescence in two genotypes of oilseed rape supplied with high (HN) or low (LN) nitrate for five days. Soluble proteins were extracted from Ténor or Samouraï cotyledons after 0 and five days of HN (3.75 mM NO_3_^−^) or LN (0.375 mM NO_3_^−^) treatment. Labeling of protease activity was carried out with MV201 (specific fluorescent probe for PLCPs, pH 5.5, 4 h labeling). After SDS-PAGE, the fluorescence was detected with a scanner (**A**) Ténor; (**B**) Samouraï. As a control, Mix refers to a mix of 0 and five day extracts (HN and LN) in the presence of MV201, NPC (no probe control) refers to the same mix in the absence of MV201, and E-64 refers to the same mix in the presence of MV201 and E-64 (specific inhibitor of PLCPs). The total amount of input proteins after incubation is represented by protein gels stained with Coomassie brilliant blue (cbb). Positions of active proteases are represented by black arrowheads. The white square and arrowhead indicate new activity during senescence in Ténor but not in Samouraï. PLCP global activity as characterized by fluorescence intensity was calculated with Image J^®^ (**C**) software. The gels are representative of three biological replicates. Gel stained with Coomassie brilliant blue (cbb) shows the RuBisCO large sub-unit (RBCL) and the decline of this major protein indicated that proteolysis and N remobilization are induced. Vertical bars indicate ± SD of the mean (*n* = 3). Different letters indicate statistical differences (*p* < 0.05, ANOVA, Newman-Keuls test).

Using the labeling of CPs with MV201, it was observed that the total specific PLCP activity was significantly increased between 0 and five days, especially under the LN condition for both genotypes (2.6 fold for Ténor and 2.4 fold for Samouraï; Figure 4B). Indeed, activities at ~40 and ~30 kDa increased during senescence between D0 and D5, while a new activity appeared at ~25 kDa under LN conditions in Ténor and Samouraï cotyledons (Figure 4A). Under LN supply, Ténor was characterized by the appearance of a new proteolytic activity at ~35 kDa not present in Samouraï (Figure 4A). The increase in PLCP activities was significantly correlated with the decrease in soluble proteins observed in Figure 1C (r = −0.827; *p*-value < 0.0001). It was already shown that several CPs were expressed in cotyledons. Indeed, genes encoding CPs were up-regulated during the development of cotyledons of soybean and common bean [44,45]. Further, the expression of γ and αVPEs (vacuolar processing enzymes) has been shown 17 days after germination in *Arabidopsis thaliana* cotyledons [46], while in upland cotton, cysteine proteases accumulate to high levels only in the yellowing cotyledons [47]. In leaves of oilseed rape, many PLCP activities increased during senescence, especially in response to nitrate limitation [29]. Additionally, a recent study showed that Ténor was characterized by higher total PLCP activity than Samouraï, and this was related to the appearance of new cysteine protease activities (RD21-like, RD19-like, SAG12-like, cathepsin-B, XBCP3-like, and aleurain-like proteases) [35]. Interestingly, as in senescent leaves, senescing cotyledons of Ténor presented additional CP activity compared with Samouraï.

In order to identify labeled CPs (Figure 4), an activity-dependent labeling with DCG04 followed by a pull-down of biotinylated proteins was performed on cotyledons of Ténor after five days of LN treatment (Figure 3, Table 1 and Table S2). The DCG04-labeling allowed the detection of four bands of active PLCPs between 37 and 25 kDa (Figure 3). Five active PLCPs were identified by LC-MS/MS (Table 1 and Table S2) and corresponded to four RD21-like proteases and one SAG12-like protease according to the classification of Richau et al. (2012) [37] (Table 1 and Table S2). All of these active proteases were also identified during leaf senescence under LN treatment in oilseed rape [29].

Moreover, activities at ~35 kDa in Ténor compared with Samouraï were associated with three different RD21-like proteases (BnaA06g36920D [*Brassica napus*]/A0A078G7A3; BnaA10g05390D [*Brassica napus*]/A0A078EXH0; BnaA08g04080D [*Brassica napus*]/A0A078FVG4). Interestingly, two of these (BnaA10g05390D [*Brassica napus*]/A0A078EXH0; BnaA08g04080D [*Brassica napus*]/A0A078FVG4) were also identified during the sequential leaf senescence of oilseed rape induced with N limitation in Ténor but not in Samouraï [35]. However, the induction of specific CP activity was observed in senescing cotyledons of Ténor in contrast to Samouraï. Interestingly, the same induced-active proteases were characteristic of Ténor during both cotyledon and leaf senescence and were associated with the higher N remobilization efficiency of Ténor than Samouraï during sequential leaf senescence.

These results showed that, even if there is no physiological difference between genotypes during the senescence of cotyledons, the PLCPs associated with the high leaf N remobilization efficiency in Ténor are found at an early stage of development. Unlike SPs, the study of CP activities at the cotyledon stage seems to be a promising way to distinguish between genotypes with contrasting proteolysis machinery and N remobilization efficiency. Furthermore, to confirm these results, it will be necessary to undertake a large-scale screening of CP activities during cotyledon senescence in many genotypes characterized by contrasting N remobilization efficiency at the leaf level.

### 3.2. Phytohormonal Regulation of Protease Activity during Senescence in Cotyledons

Based on the results presented above, cotyledons may provide an excellent experimental system to study senescence processes and their regulation systems, similar to other species such as upland cotton, soybean, and cucumber [47,48,49]. Moreover, in a recent study [35], we reported that during the leaf senescence associated with N limitation, the high N remobilization efficiency of the genotype Ténor was characterized by a higher senescence-promoting hormonal ratio ([SA] + [ABA])/([CKs]) that was correlated with higher proteolysis and protease activities than Samouraï. In order to validate the putative role of SA and ABA in the regulation of protease activity during senescence, infiltrations of exogenous SA or ABA were carried out on mature cotyledons of Ténor and physiological and proteolysis modifications were studied three days after infiltration.

#### 3.2.1. Physiological and Proteolysis Modifications during Cotyledon Senescence Associated with SA and ABA Infiltrations

##### Effects of ABA Infiltrations of Cotyledon Senescence

The promoter role of ABA during senescence has been demonstrated many times in various species [19,50,51,52,53,54]. Indeed, many genes involved in the synthesis of the ABA or ABA-related signal are overexpressed during senescence [52], while the endogenous levels of ABA increase during senescence in several species such as *Arabidopsis thaliana* or maize [51,52]. In oilseed rape, in response to N limitation, endogenous ABA also significantly increases during the leaf senescence of the genotype Ténor compared with Samouraï [35].

**Figure 5:**
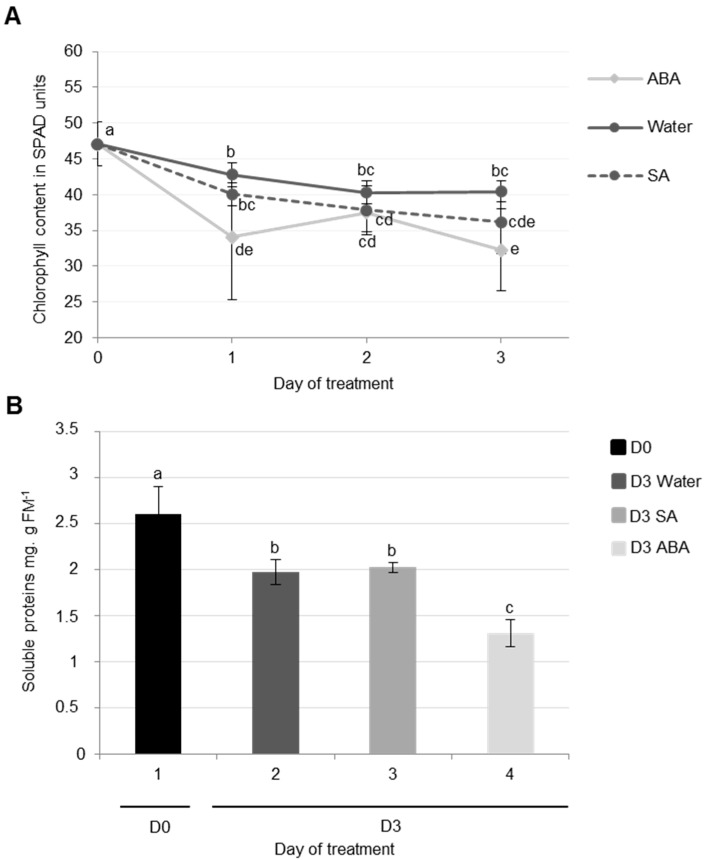
Chlorophyll (**A**) and soluble protein (**B**) contents of cotyledons in oilseed rape (cv. Ténor) at 0 or three days after water, SA, or ABA infiltrations. Plants (15 days old) were infiltrated with 1 mL of water, salicylic acid (SA, 500 μM), or abscisic acid (ABA, 50 μM). The infiltration was performed on the abaxial surface of the cotyledons with a needleless syringe by simple pressure. Vertical bars indicate ± SD of the mean (*n* = 3). Different letters indicate statistical differences (*p* < 0.05, ANOVA, Newman-Keuls test).

The results showed that chlorophyll content strongly decreased during cotyledon senescence three days after infiltration with ABA (−31%) compared with the control (water infiltration, −14%) (Figure 5A). It was also demonstrated that the exogenous application of ABA (75 μM) resulted in a decrease in chlorophyll content and the repression of chloroplast genes transcription in *Hordeum vulgare* leaves [55]. Moreover, several mutants of *Arabidopsis thaliana* that are insensitive to ABA have presented a stay-green phenotype without the degradation of chlorophylls [53,54].

**Figure 6:**
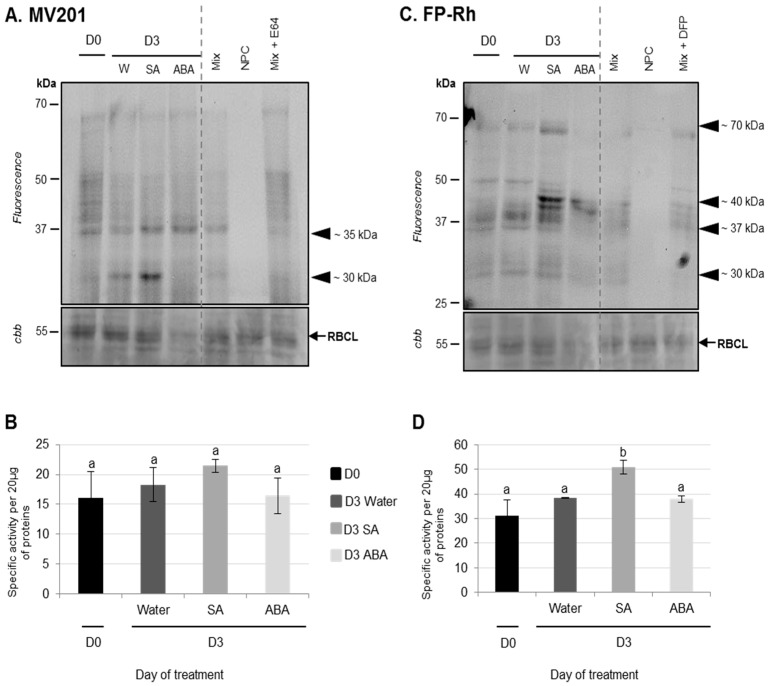
PLCP and SH activities of cotyledons of oilseed rape (cv. Ténor) at 0 or three days after water, SA, or ABA infiltrations. Plants (15 days old) were infiltrated with 1 mL of water, salicylic acid (SA, 500 μM), or abscisic acid (ABA, 50 μM). Soluble proteins of cotyledons were extracted from Ténor plants after 0 and three days of infiltration treatments. Labeling of protease activity was carried out with MV201 or FP-Rh (specific fluorescent probes of PLCPs and SHs respectively, pH 5.5, 4 h labeling for MV201 and pH 7.5; 1 h labeling for FP-Rh). After SDS-PAGE, the fluorescence was detected with a scanner ((**A**) PLCPs; (**C**) SHs). As a control, Mix refers to a mix of 0 and five day extracts (HN and LN) in the presence of MV201 or FP-Rh. NPC (no probe control) refers to the same mix in the absence of MV201 or FP-Rh. In addition, competition experimentation was performed with the same mix in the presence of E-64 or DFP, corresponding to specific inhibitors of PLCPs or SHs, respectively. Positions of active proteases are represented by black arrowheads. PLCP or SH global activity as characterized by the fluorescence intensity was calculated with Image J^®^ software (**B**) PLCPs; (**D**) SHs). The presented gels are representative of three biological replicates. Gel stained with Coomassie brilliant blue (cbb) shows the RuBisCO large sub-unit (RBCL) and the decline of this major protein indicated that proteolysis and N remobilization are induced. Vertical bars indicate ± SD of the mean (*n* = 3). Different letters indicate statistical differences (*p* < 0.05, ANOVA, Newman-Keuls test).

Soluble protein contents decreased during the senescence of cotyledons after three days, particularly after ABA infiltration (−49% vs. only −24% in control) (Figure 5B). This high rate of proteolysis in response to ABA is mainly due to the strong degradation of the large sub-unit of RuBisCO (Figure 6). These observations are consistent with the fact that an exogenous application of ABA induced a diminution of protein content, particularly RuBisCO in rice leaves [56].

Using the labeling of CPs with MV201, it was observed that while the total specific PLCPs remained stable for three days regardless of the infiltration (Figure 6B), PLCP activities at ~35 kDa were increased in response to ABA infiltrations compared to water infiltration (Figure 6A). It was already demonstrated that the application of ABA could provoke an enhancement of protease activities in rice leaves [56]. Using the labeling of SHs with Fp-Rh probe, it was observed that the total specific SHs remained stable for three days after water or ABA infiltration and that no specific SH was increased after ABA infiltration (Figure 6C,D).

##### Effects of SA Infiltrations on Cotyledon Senescence in Ténor

Salicylic acid (SA) has a role in senescence acceleration [20,23,57,58,59]. Indeed, during leaf senescence in *Arabidopsis thaliana*, the concentration of SA increases [23] and many genes involved in the synthesis of SA are induced [58].

Compared to ABA infiltration, the results showed that the infiltration of exogenous SA into cotyledons did not induce physiological modifications associated with senescence. Indeed, the decreases in chlorophyll and soluble protein contents in SA-infiltrated cotyledons were not significantly different to the control (water infiltration, Figure 5A,B). Using the labeling of CPs with MV201, it appears that the total specific PLCPs remained stable regardless of the infiltration (Figure 6B). Nevertheless, the activity of PLCPs at ~35 kDa was increased by SA infiltration and a new strong CP activity at ~30 kDa was specifically observed only after SA infiltration (Figure 6A). These results were consistent with several studies. Indeed, an exogenous treatment with SA in *Arabidopsis thaliana* enhanced the expression of *SAG* genes [57,59], including genes encoding VPEs (αVPE and γVPE) [20]. Further, Morris et al. (2000) [59] demonstrated that a defect in the synthesis of SA in *Arabidopsis thaliana* delayed senescence and repressed the gene expression of *SAG12* (encoding a cysteine protease). It was also reported that SA acted as a repressor of cystatin expression (which is one of the main CP inhibitors) [60].

In addition, using the labeling of SHs with Fp-Rh, it was observed that the total specific activities of SHs were increased three days after SA infiltration (+62%) compared to water infiltration (+22%) (Figure 6D). This was particularly associated with the increase in SH activities at ~70 kDa and ~40 kDa (Figure 6C). The up-regulation of genes encoding for serine proteases (including ATP-dependent Clp and a putative serine protease) was already shown in seedling leaves of sorghum after an SA application [61]. As reported in the leaf senescence of oilseed rape or other species, it appears that ABA and SA are able to modulate the proteolysis processes observed during cotyledon senescence, suggesting that ABA and SA may have a generic role in the regulation of protein remobilization of the different kinds of senescence.

## 4. Conclusions

Regardless of the genotype, this study demonstrated that the senescence of cotyledons in oilseed rape was characterized by a decrease in chlorophyll and soluble protein contents correlated with an increase in SH and CP activities, particularly in response to N limitation. These modifications and many identified CPs/SPs were also observed during the leaf senescence of oilseed rape [9,29,62], meaning that cotyledons may provide a promising experimental system to study the proteolysis machinery associated with senescence processes, as well as the regulation of senescence.

Although Ténor, in comparison to Samouraï, was characterized by a higher N remobilization efficiency during the sequential leaf senescence associated with N limitation [8,9], no differences were observed between the genotypes for yellowing or protein content during cotyledon senescence. However, two RD21-like proteases characteristic of the higher protein degradation of Ténor were detected in leaves and cotyledons during senescence. That is why, unlike SPs, the study of PLCP activities by ABPP methods seems to be a promising tool to undertake the large scale characterization of genotypes under contrasting N remobilization efficiencies as early as the cotyledon stage of development.

Using a cotyledon model with the genotype Ténor, it was demonstrated that the infiltration of ABA decreases the chlorophyll and soluble protein contents and induces several types of PLCP activities, whereas the infiltration of SA does not induce modifications of physiological senescence parameters but increases the activities of two PLCPs and several SHs. The specific impacts of ABA and SA on the proteolysis machinery involved in cotyledon senescence are also reported when leaf senescence is induced by N limitation, a treatment that leads to increases in the ABA and SA contents in senescing leaves of Ténor [35]. Overall, the data suggests that the study of protease activities during cotyledon senescence is a promising experimental model system to investigate the genotypic variability for contrasting N remobilization efficiency and the regulation of proteolysis machinery involved in a high N remobilization efficiency.

## Figures and Tables

**Table 1:** Identifications of CPs and SPs labeled with the biotin-tagged probe DCG04 and FP, respectively, in senescing cotyledons of oilseed rape (cv. Ténor) after five days of nitrate limitation by LC-MS/MS. The assigned protein with the best match is provided alongside the Uniprot accession number (if available) or NCBI/GenBank accession number. Proteases were classified according to Richau et al. (2012) [37] for CPs and MEROPS database for SPs.

Protein Accession No. [*Brassica Napus*]/Uniprot or NCBI Accession No.	Classification
*Cysteine Proteases*	
BnaA08g04080D [*Brassica napus*]/A0A078FVG4	RD21-like
BnaA10g05390D [*Brassica napus*]/A0A078EXH0	RD21-like
PREDICTED: cysteine proteinase RD21a [*Brassica napus*]/XP_013718810	RD21-like
BnaA06g36920D [*Brassica napus*]/A0A078G7A3	RD21-like
senescence-specific cysteine protease [*Brassica napus*]/AAD53011	SAG12-like
*Serine Proteases*	
PREDICTED: serine carboxypeptidase-like 49 [*Brassica napus*]/XP_013676539	S10
BnaA06g18620D [*Brassica napus*]/A0A078EDE5	S10
BnaA01g06330D [*Brassica napus*]/A0A078GRW5	S10
BnaA10g23100D [*Brassica napus*]/A0A078BZ09	S10
PREDICTED: serine carboxypeptidase-like 35 [*Brassica napus*]/XP_013701943	S10
BnaCnng56420D [*Brassica napus*]/A0A078JMY7	S10
PREDICTED: serine carboxypeptidase-like 29 [*Brassica napus*]/XP_013750696	S10
BnaA04g07190D [*Brassica napus*]/A0A078HQ25	S10
PREDICTED: serine carboxypeptidase-like 29 [*Brassica napus*]/XP_013730766	S10
BnaA08g12880D [*Brassica napus*]/A0A078GF58	S10
BnaA04g16130D [*Brassica napus*]/A0A078GVN3	S10
PREDICTED: serine carboxypeptidase-like 20 [*Brassica napus*]/XP_013696030	S10
PREDICTED: subtilisin-like protease SBT1.7 [*Brassica napus*]/XP_013654072	Subtilisin S8
PREDICTED: protease Do-like 1, chloroplastic [*Brassica napus*]/XP_013644609	Deg protease

## Supplementary Materials

The following are available online www.mdpi.com/2227-7382/5/4/29/s1. Table S1: Identified serine hydrolases labeled with FP- biotin in senescent cotyledons of oilseed rape (cv. Ténor) after five days of LN treatment; Table S2: Identified cysteine proteases labeled with DCG04 in senescent cotyledons of oilseed rape (cv. Ténor) after five days of LN treatment; LC/MS data for active serine and cysteine proteases are given in a supplemental excel file.

## Abbreviations

AALP	aleurain-like protease
ABA	abscisic acid
CP	cysteine protease
HN	high nitrate
LN	low nitrate
PLCP	papain-like cysteine protease
RuBisCO	ribulose-1,5-biphosphate carboxylase/oxygenase
SA	salicylic acid
SH	serine hydrolase
SP	serine protease
VPE	vacuolar processing enzyme

## References

[1] Carré P., Pouzet A. (2014). Rapeseed market, worldwide and in Europe. Oilseeds Fats Crop. Lipids.

[2] Rathke G.W., Christen O., Diepenbrock W. (2005). Effects of nitrogen source and rate on productivity and quality of winter oilseed rape (*Brassica napus* L.) grown in different crop rotations. Field Crops Res..

[3] Schjoerring J.K., Bock J.G.H., Gammelvind L., Jensen C.R., Mogensen V.O. (1995). Nitrogen incorporation and remobilization in different shoot components of field-grown winter oilseed rape (*Brassica napus* L.) as affected by rate of nitrogen application and irrigation. Plant Soil.

[4] Malagoli P., Laîné P., Rossato L., Ourry A. (2005). Dynamics of nitrogen uptake and mobilization in field-grown winter oilseed rape (*Brassica napus*) from stem extension to harvest II. An 15N-labelling-based simulation model of N partitioning between vegetative and reproductive tissues. Ann. Bot..

[5] Malagoli P., Laîné P., Rossato L., Ourry A. (2005). Dynamics of nitrogen uptake and mobilization in field-grown winter oilseed rape (*Brassica napus*) from stem extension to harvest: I. Global N flows between vegetative and reproductive tissues in relation to leaf fall and their residual N. Ann. Bot..

[6] Gombert J., Etienne P., Ourry A., Le Dily F. (2006). The expression patterns of *SAG12/Cab* genes reveal the spatial and temporal progression of leaf senescence in *Brassica napus* L. with sensitivity to the environment. J. Exp. Bot..

[7] Avice J.C., Etienne P. (2014). Leaf senescence and nitrogen remobilization efficiency in oilseed rape (*Brassica napus* L.). J. Exp. Bot..

[8] Girondé A., Poret M., Etienne P., Trouverie J., Bouchereau A., Le Cahérec F., Leport L., Orsel M., Niogret M.F., Deleu C. (2015). A profiling approach of the natural variability of foliar N remobilization at the rosette stage gives clues to understand the limiting processes involved in the low N use efficiency of winter oilseed rape. J. Exp. Bot..

[9] Girondé A., Poret M., Etienne P., Trouverie J., Bouchereau A., Le Cahérec F., Leport L., Niogret M.F., Avice J.C. (2016). A comparative study of proteolytic mechanisms during leaf senescence of four genotypes of winter oilseed rape highlighted relevant physiological and molecular traits for NRE improvement. Plants.

[10] Bouchet A.S., Laperche A., Bissuel-Belaygue C., Snowdon R., Nesi N., Stahl A. (2016). Nitrogen use efficiency in rapeseed. A review. Agron. Sustain. Dev..

[11] Krupinska K., Mulisch M., Hollmann J., Tokarz K., Zschiesche W., Kage H., Humbeck K., Bilger W. (2012). An alternative strategy of dismantling of the chloroplasts during leaf senescence observed in a high-yield variety of barley. Physiol. Plant.

[12] Kim J., Woo H.R., Nam H.G. (2016). Toward systems understanding of leaf senescence: An integrated multi-omics perspective on leaf senescence research. Mol. Plant.

[13] Diaz-Mendoza M., Velasco-Arroyo B., Santamaria M.E., González-Melendi P., Martinez M., Diaz I. (2016). Plant senescence and proteolysis: Two processes with one destiny. Genet. Mol. Biol..

[14] Thomas H., Stoddart J. (1980). Leaf senescence. Annu. Rev. Plant Physiol..

[15] Wu X.Y., Kuai B.K., Jia I.Z., Jing H.C. (2012). Regulation of leaf senescence and crop genetic improvement. J. Int. Plant Biol..

[16] Gregersen P.L., Cutelic A., Boschian L., Krupinska K. (2013). Plant senescence and crop productivity. Plant Mol. Biol..

[17] Guo Y., Gan S.S. (2005). Leaf senescence: Signals, execution, and regulation. Curr. Top. Dev. Biol..

[18] Kusaba M., Tanaka A., Tanaka R. (2013). Stay-green plants: What do they tell us about the molecular mechanism of leaf senescence?. Photosynth. Res..

[19] Lim P.O., Kim H.J., Nam H.G. (2007). Leaf senescence. Annu. Rev. Plant Biol..

[20] Jibran R., Hunter D.A., Dijkwel P.P. (2013). Hormonal regulation of leaf senescence through integration of developmental and stress signals. Plant Mol. Biol..

[21] Sarwat M., Naqvi A.R., Ahmad P., Ashraf M., Akram N.A. (2013). Phytohormones and microRNAs as sensors and regulators of leaf senescence: Assigning macro roles to small molecules. Biotechnol. Adv..

[22] Zhang H., Zhou C. (2013). Signal transduction in leaf senescence. Plant Mol. Biol..

[23] Khan M., Rozhon W., Poppenberger B. (2014). The role of hormones in the aging of plants—A mini-review. Gerontology.

[24] Guo Y., Gan S.S. (2014). Translational researches on leaf senescence for enhancing plant productivity and quality. J. Exp. Bot..

[25] Mueller-Roeber B., Balazadeh S. (2014). Auxin and its role in plant senescence. J. Plant Growth Regul..

[26] Kant S., Burch D., Badenhorst P., Palanisamy R., Mason J., Spangenberg G. (2015). Regulated expression of a cytokinin biosynthesis gene IPT delays leaf senescence and improves yield under rainfed and irrigated conditions in canola (*Brassica napus* L.). PLoS ONE.

[27] Zeng X.F., Zhao D.G. (2016). Expression of IPT in Asakura-sanshoo (*Zanthoxylum piperitum* (L.) DC. f. inerme Makino) alters tree architecture, delays leaf senescence, and changes leaf essential oil composition. Plant Mol. Biol. Rep..

[28] Desclos-Théveniau M., Coquet L., Jouenne T., Etienne P. (2014). Proteomic analysis of residual proteins in blades and petioles of fallen leaves of *Brassica napus*. Plant Biol..

[29] Poret M., Chandrasekar B., van der Hoorn R.A.L., Avice J.C. (2016). Characterization of senescence-associated protease activities involved in the efficient protein remobilization during leaf senescence of winter oilseed rape. Plant Sci..

[30] Roberts I.N., Caputo C., Criado M.V., Funk C. (2012). Senescence-associated proteases in plants. Physiol. Plant..

[31] Bhalerao R., Keskitalo J., Erlandsson R., Björkbacka H., Birve S.J., Karlsson J., Gardeström P., Gustafsson P., Lundeberg J., Jansson S. (2003). Gene expression in autumn leaves. Plant Physiol..

[32] Diaz I., Martinez M., Rawlings N.D., Salvesen G. (2013). Plant C1A cysteine peptidases in germination and senescence. Handbook of Proteolytic Enzymes.

[33] Diaz-Mendoza M., Velasco-Arroyo B., Gonzalez-Melendi P., Martinez M., Diaz I. (2014). C1A cysteine protease-cystatin interactions in leaf senescence. J. Exp. Bot..

[34] Kidric M., Kos J., Sabotic J. (2014). Protease and their endogenous inhibitors in the plant response to abiotic stress. Bot. Serbica.

[35] Poret M. (2016). Caractérisation de la Machinerie Protéolytique Associée à une Remobilisation Efficiente de L’azote Pendant la Sénescence Dans le but D’optimiser L’efficience D’usage de L’azote Chez le Colza (*Brassica napus* L.). Ph.D. Thesis.

[36] Patricelli M.P., Giang D.K., Stamp L.M., Burbaum J.J. (2001). Direct visualization of serine hydrolase activities in complex proteomes using fluorescent active site-directed probes. Proteomics.

[37] Richau K.H., Kaschani F., Verdoes M., Pansuriya T.C., Niessen S., Stüber K., Colby T., Overkleeft H.S., Bogyo M., van der Hoorn R.A.L. (2012). Subclassification and biochemical analysis of plant papain-like cysteine proteases displays subfamily-specific characteristics. Plant Physiol..

[38] Bradford M.M. (1976). A rapid and sensitive method for the quantitation of microgram quantities of protein utilizing the principle of protein-dye binding. Anal. Biochem..

[39] Chandrasekar B., Colby T., Emran Khan Emon A., Jiang J., Hong T.N., Villamor J.G., Harzen A., Overkleeft H.S., van der Hoorn R.A.L. (2014). Broad-range glycosidase activity profiling. Mol. Cell. Proteom..

[40] Blum H., Beier H., Gross H.J. (1987). Improved silver staining of plant proteins, RNA and DNA in polyacrylamide gel. Electrophoresis.

[41] Ghosh S., Mahoney S.R., Penterman J.N., Peirson D., Dumbroff E.B. (2001). Ultrastructural and biochemical changes in chloroplasts during *Brassica napus* senescence. Plant Physiol. Biochem..

[42] Desclos M., Etienne P., Coquet L., Jouenne T., Bonnefoy J., Segura R., Reze S., Ourry A., Avice J.C. (2009). A combined ^15^N tracing/proteomics study in *Brassica napus* reveals the chronology of proteomics events associated with N remobilisation during leaf senescence induced by nitrate limitation or starvation. Proteomics.

[43] Kim D.J. (2004). Study of cotyledon senescence in cucumber (*Cucumis sativus* L.) based on expressed sequence tags and gene expression. J. Plant Biol..

[44] Yamauchi D. (2007). Hormonal regulation of the expression of cysteine proteinase genes in germinated cotyledons of common bean seeds. Plant Biotechnol..

[45] Brown A.V., Hudson K.A. (2015). Developmental profiling of gene expression in soybean trifoliate leaves and cotyledons. BMC Plant Biol..

[46] Kinoshita T., Yamada K., Hiraiwa N., Kondo M., Nishimura M., Hara-Nishimura I. (1999). Vacuolar processing enzyme is up-regulated in the lytic vacuoles of vegetative tissues during senescence and under various stressed conditions. Plant J..

[47] Shen F., Yu S., Xie Q., Han X., Fan S. (2006). Identification of genes associated with cotyledon senescence in upland cotton. Chin. Sci. Bull..

[48] Ling J.Q., Kojima T., Shiraiwa M., Takahara H. (2003). Cloning of two cysteine proteinase genes, CysP1 and CysP2, from soybean cotyledons by cDNA representational difference analysis. Biochim. Biophys. Acta.

[49] Bangerth K.F. (2015). Basipetal auxin versus acropetal cytokinin transport, and their interaction with NO_3_ fertilisation in cotyledon senescence and sink: Source relationships in cucumber (*Cucumis sativus* L.). Plant Biol..

[50] Weaver L.M., Gan S., Quirino B., Amasino R.M. (1998). A comparison of the expression patterns of several senescence-associated genes in response to stress and hormone treatment. Plant Mol. Biol..

[51] He P., Osaki M., Takebe M., Shinano T., Wasaki J. (2005). Endogenous hormones and expression of senescence-related genes in different senescent types of maize. J. Exp. Bot..

[52] Breeze E., Harrison E., McHattie S., Hughes L., Hickman R., Hill C., Kiddle S., Kim Y.S., Penfold C.A., Jenkins D. (2011). High-resolution temporal profiling of transcripts during Arabidopsis leaf senescence reveals a distinct chronology of processes and regulation. Plant Cell.

[53] Lee I.C., Hong S.W., Whang S.S., Lim P.O., Nam H.G., Koo J.C. (2011). Age-dependent action of an ABA-inducible receptor kinase, RPK1, as a positive regulator of senescence in Arabidopsis leaves. Plant Cell Physiol..

[54] Zhang K., Gan S.S. (2012). An abscisic acid-AtNAP transcription factor-SAG113 protein phosphatase 2C regulatory chain for controlling dehydration in senescing Arabidopsis leaves. Plant Physiol..

[55] Yamburenko M.V., Zubo Y.O., Vanková R., Kusnetsov V.V., Kulaeva O.N., Börner T. (2013). Abscisic acid represses the transcription of chloroplast genes. J. Exp. Bot..

[56] Fukayama H., Abe R., Uchida N. (2010). SDS-dependent proteases induced by ABA and its relation to Rubisco and Rubisco activase contents in rice leaves. Plant Physiol. Biochem..

[57] Buchanan-Wollaston V., Page T., Harrison E., Breeze E., Lim P.O., Nam H.G., Lin J.F., Wu S.H., Swidzinski J., Ishizaki K. (2005). Comparative transcriptome analysis reveals significant differences in gene expression and signalling pathways between developmental and dark/starvation-induced senescence in Arabidopsis. Plant J..

[58] Van der Graaff E., Schwacke R., Schneider A., Desimone M., Flugge U.I., Kunze R. (2006). Transcription analysis of Arabidopsis membrane transporters and hormone pathways during developmental and induced leaf senescence. Plant Physiol..

[59] Morris K., Mackerness S.A.H., Page T., John C.F., Murphy A.M., Carr J.P., Buchanan-Wollaston V. (2000). Salicylic acid has a role in regulating gene expression during leaf senescence. Plant J..

[60] Doares S.H., Narvaez-Vasquez J., Conconi A., Ryan C.A. (1995). Salicylic acid inhibits synthesis of proteinase inhibitors in tomato leaves induced by systemin and jasmonic acid. Plant Physiol..

[61] Salzman R.A., Brady J.A., Finlayson S.A., Buchanan C.D., Summer E.J., Sun F., Klein P.E., Klein R.R., Pratt L.H., Cordonnier-Pratt M.M. (2005). Transcriptional profiling of sorghum induced by methyl jasmonate, salicylic acid, and aminocyclopropane carboxylic acid reveals cooperative regulation and novel gene response. Plant Physiol..

[62] Girondé A., Etienne P., Trouverie J., Bouchereau A., Le Caherec F., Leport L., Orsel M., Niogret M.F., Nesi N., Carole D. (2015). The contrasting N management of two oilseed rape genotypes reveals the mechanisms of proteolysis associated with leaf N remobilization and the respective contributions of leaves and stems to N storage and remobilization during seed filling. BMC Plant Biol..

